# Academy of Stomatology

**Published:** 1905-03

**Authors:** 


### ACADEMY OF STOMATOLOGY.

A REGULAR monthly meeting of the Academy of Stomatology of Philadelphia was held at its rooms, 1737 Chestnut Street, on the evening of Tuesday, March 1, 1904, the President, Dr. L. Foster Jack, in the chair.

A paper entitled "Some Studies in Occlusion"[1] was read by Dr. Edward H. Angle, of St. Louis.

(For Dr. Angle's paper, see page 165.)

[1] This paper of Dr. Angle's was extensively illustrated by the lantern, but as the author has not furnished the necessary photographs, it is presumed he regards the text as sufficiently clear without them. Most of these have been previously published in Dr. Angle's articles on related subjects. They were all to prove that the full complement of teeth is necessary to the best occlusion and best harmony of facial lines in all cases, and that extraction, according to the essayist, is as disgraceful as it is unnecessary and behind the times.—ED.

DISCUSSION.

*Dr. M. H. Cryer.*—Dr. Angle speaks of moving a tooth backward as if it were an easy thing to do. I believe my friend, Dr. Head, thinks the same. I do not believe that the lower molar can be pushed backward. It can be moved forward, but not backward. The anatomy of the tissues allows everything to go forward, both in the upper and lower jaws, but I do not believe the backward movement can be as easily accomplished as Dr. Angle believes. He states that Nature makes a certain set of teeth for one face and another for another. That is true, but she makes the greatest mistakes in doing it. We would not be here as dentists if it were not to correct Nature. We live in houses to protect ourselves from the cold winters we have. We cross the stormy deep with perfect safety in spite of Nature. There is only one thing yet which we cannot control,—the volcano, such as that at Martinique; but the time will come when we will even harness that as we are doing Niagara, and utilize it.

I have a specimen here of a small mouth of a white person. See the amount of tooth tissue that is in that specimen. There are not only sixteen, but eighteen teeth in the upper jaw. In the small mouth that is uncommon, but Nature has made it. Therefore these teeth ought to be brought into line. If we correct that, we push the teeth forward and carry the lip outward.

Here is a specimen of a much larger mouth. The teeth are almost like deciduous teeth. They are small, with spaces between. This is a lower jaw, and you see the profile that we have. These teeth pass outward, carrying the lips, making the face, as Dr. Talbot, of Chicago, would say, that of a degenerate. The incisors and laterals are a little out of position. Dr. Angle here would say widen the arch. There are cases with which we must work according to the material. I believe there are cases in which we should extract a tooth or four teeth rather than keep them in. I have a patient who has been in the hands of an enthusiastic man following the method advocated by Dr. Angle, and I fear that these lips are going to protrude and that bone will not be developed to support those teeth either upper or lower. These teeth at the present time ought to be almost perpendicular.

The jaw that Dr. Angle showed was not a typical one. Here you have a typical jaw, and not one of a negro. This is a true and typical occlusion.

If I have a patient whose appearance I can improve by extracting four bicuspids and getting the teeth into line, I am going to do it, no matter what Nature has done. I have slides here that I would delight to show where that has been done with improvement to the face.

*The President.*—You say that you cannot move a tooth backward; when you extract bicuspids, how do you fill the space?

*Dr. Cryer.*—I said that molars could not be moved backward. You must make some fixed point to prove that you have moved the molars backward.

*Dr. S. M. Weeks.*—I feel very much embarrassed in speaking on the subject after the remarks that have been made. I have been very much interested in the paper by Dr. Angle, and it can call from me nothing but commendation, though the paper speaks for itself and needs no commendation of mine.

The case spoken of by Dr. Cryer is a patient whom I have had for treatment. I am sorry that he did not have the slides to show this evening. I have the photographs. The patient is twelve years of age. While there may be, as Dr. Cryer has said, a suggestion of too much prominence in the lips at the present time, I believe with Dr. Angle that, with the general development of the bones and soft parts during the coming years, this will be overcome; but only time can settle that fact.

*Dr. Cryer.*—For Dr. Angle's method of moving teeth and that of Dr. Weeks I have the highest commendation. I concede to Dr. Angle all the way, excepting in those few cases I speak of, and I think I am right; I do not know positively. I am sorry that I cannot stay and listen to Dr. Angle's reply, and am sorry that he is going away so soon. I most certainly appreciate what he has done in coming here, and he is certainly doing a great deal of good work.

*Dr. J. Head.*—I can only say from my personal experience that in hearing Dr. Angle's paper to-night I have been moved on one side with admiration and on the other with self-condemnation to think that I have done so much harm in the past by not following the lines he has laid down.

Regarding Dr. Cryer's remarks about the mistakes of Nature and the mind of man fighting Nature, it seems to me that the mind of man has a pretty big contract on its hand if it attempts to remake Nature and pick out individual points wherein she has made mistakes. When I was down in Washington last week I heard it declared that the teeth were not necessary for mastication; in fact, that mastication was not necessary for the human health, that frequently people who lost their teeth were much better without them. I also heard that the temporary teeth frequently were not necessary. I have frequently heard that we may extract teeth for the sake of improving the contour of the face. It is a serious thing for us to decide, when the face is yet undeveloped, whether we are going to improve that contour. How can we tell what that face is going to be at the age of thirty? What Dr. Cryer says may be so. There may be cases in which the four molars will change the plan of articulation to a certain extent. These conditions have to be met separately. I do think that too much praise cannot be given to Dr. Angle for giving us a scientific classification. When we find that within the Angle classification we can go forward with absolute safety as to an ultimate outcome, I think we should act on his lines. A tooth put into absolute occlusion will stay in occlusion, and a tooth that is not in occlusion will go into some other position. So much do I believe in the care and preservation of the permanent teeth that I am now instructing mothers to see that with their children of three or five years the silk floss is used between the teeth each day and the teeth carefully brushed afterwards. When the first permanent molars erupt, I watch the child to see that the cuspids come in with proper and normal occlusion. Sometimes the mother thinks the child does not need any care until it is seven or eight years of age. We frequently then find that harm has already been done; that the first molars have slipped out of occlusion. I think a good plan is to take those temporary molars that are going to be lost and put bands on them, and so arrange the cuspids that the child will be compelled to bite normally. In that way, by the time the temporary molars are lost the occlusion will be correct.

*Dr. H. E. Roberts.*—I wish to speak of a young man who had been under my care since the time he was six years of age. Six years ago he went from under that care, and at that time I believed his teeth to be in perfect occlusion. I saw him lately at twenty-one years of age. At fifteen his teeth were apparently in perfect occlusion; at twenty-one his lower jaw protrudes the full width of the third molar beyond the uppers. I think this Dr. Angle calls the third class. I never have seen a more pronounced case.

The elongation of the molars to correct the bite of the incisors, spoken of by Dr. Angle to-night, I advocated at a meeting at which Dr. William H. Trueman was present, some twenty years ago, and Dr. Trueman has used that method himself. Bring all the bite on the front teeth or the incisors, and the molars will, as Dr. Angle says, in from three to five months' time elongate; you can bring them up almost as much as you choose.

*Dr. W. H. Trueman.*—During the time I have been in dentistry I have kept my eyes pretty wide open, and I think I have seen some things. When I began I was quite enthusiastic about orthodontia. I think I was very successful. I know I gained considerable reputation with my work and put thousands of dollars into my pocket. As time went on I have seen the result of some of these operations, and I am not now as enthusiastic as before. The question is now asked me by a patient, "Can these teeth be made regular?" I say, "Yes." The reply is, "I shall be glad to have you undertake it." I say, "No, I refuse, because I think they are better as they are." And so I think with a great many regulating cases,—while I yield to no one in my admiration for what Dr. Angle has done,—a great deal of the work is of no real, clinical, practical value. I have watched many cases of my own and of others, and while time has gone on I have been exceedingly doubtful whether any real good has been accomplished. I would like to know what the result has been in these cases reported and illustrated after an interval of ten or fifteen years. I recall one case that illustrates very nicely what Dr. Cryer has said, in which there was a lateral curve instead of an arch. It is an easy matter to regulate such a case, and if when the tooth is brought into position it overlaps the tooth by one-sixteenth of an inch, we would say that that tooth, once brought into position, would stay there. This case I was doubtful about undertaking. The young man was eighteen years of age, and the configuration of his teeth was such as to indicate that it would be difficult to get the tooth to stay; but I succeeded in bringing it about. I put on a retaining fixture and said it would have to remain some months. After six months I removed it. Then I saw him at short intervals to observe that the tooth remained in place. It was there all right for two or three years. In two or three years more it was back where I found it. Why was that tooth moved backward? The lower teeth were in position to keep it in place. Why did it get back to its old position? We have but to consider what Dr. Cryer said,—that there is something we inherit from our remote ancestors. There is a constant pushing forward of all the teeth. In some cases it is more marked than in others. We cannot by our art go against Nature. We must remember there is constant change.

To illustrate another point. Some years ago a case was brought to the city for surgical operation and fell into the hands of some dentists. One of the central teeth had met with an accident. It was shorter than the others, and it was suggested that this could easily be brought down. I was present, and I asked whether that could be safely done? The answer was, "Oh, yes." It was at about the time that nerve-stretching came into vogue. It was stated that stretching of these tissues in young people could not produce harm. Later on I saw the case while the apparatus was in place, and still later saw it when it was pronounced a complete success. It was questioned whether the tooth had sustained any injury, and the statement was that there was only a little change in color, but no injury had been sustained. A few years later I learned that the pulp had died, the tooth abscessed, and that, in spite of all that could be done, it had to be extracted. It would have been far better had it been left alone, for in time it would have assumed a somewhat normal position. Interference with it cost the loss of a useful tooth.

In one case I had, in which the upper and lower dentures were crowded, I took casts and showed them to my preceptor. He advised me to extract the four first bicuspids and the work would be done. I did not like that. I took these casts to one of our dental associations and had them carefully examined. I was told to expand the arch. I told my preceptor, and he said, "Take out the four bicuspid teeth and the work is done." I undertook to do it. I took out the two upper bicuspids and left the lower ones in place. I expanded the arch, and brought them into position. I saw the case at intervals of thirty years. While it was very successful in so far as it brought me reputation, to me it was not successful. I had very little trouble with the upper teeth, but much with the lower, and in fact, forced them into position. I presume that by this time that jaw is almost edentulous. I would like to see these cases in which the delicate tissue is stretched ten or fifteen years afterwards, and know what the result is then, not the result immediately after the operation is done. Occlusion is a very important point; but when we secure that perfect occlusion we must ask, Will Nature permit it to remain so?

A gentleman came to Robert Arthur, first graduate of the Baltimore Dental College, some years ago with perfect upper and lower dentures which articulated and occluded nicely. Later, changes took place, not from breaking down, but from normal wear, and the occlusion had become a protrusion. The changes were so great that photographs taken ten years before would hardly be recognized as those of the individual. Nature will assert herself, and I think she will rebel in some cases of regulating which we sometimes think have been so very successful. I would suggest that Dr. Angle let us know what the result is ten or fifteen years after the work has been accomplished.

*Dr. M. I. Schamberg.*—There is no doubt but that the progressive men of dentistry will give Dr. Angle credit for the advances made in this direction. I would like to ask him whether, in moving the teeth backward for young people, the third molars, which are already crowded, are not likely to give trouble. We find to-day that the jaw scarcely accommodates them, and it is surprising how very many cases require almost surgical means for their removal. Only a person specializing in work of that kind can realize the tremendous number of wisdom-teeth that are impacted, and I was wondering whether Dr. Angle's method would influence those teeth.

In reference to the premature resorption of temporary teeth, I would like to state that I have several radiographs showing conclusively that resorption frequently takes place in the temporary teeth when the permanent are not within reach.

In regard to the habit Dr. Angle speaks of, of a patient with enlarged tonsils throwing the lower jaw forward, causing its protrusion, I would say that I have had several cases sent to me for removal of adenoids and tonsils prior to regulation, and I believe that the throat is so largely filled with tissue, the post-pharyngeal glands, and the tonsils that the patient tries to create a larger breathing space by throwing the lower jaw forward.

*Dr. Angle* (closing).—It is already late, but I would like to hear from many more. It is not often I get a chance to have people pitch into me, and I rather enjoy it. I have not much to say now, because I tried to bring out in my paper all the points that have been touched upon in the discussion.

First, in regard to Dr. Cryer's criticism. We are not looking for exceptions to rules. There are exceptions to all rules, and it is also said that they prove the rule. Now the exceptions that Dr. Cryer has mentioned do not prove anything. It is true that we now and then find freaks, but they certainly should not be taken as a basis for rules to govern normal people. I do not know of a man on this earth who is more fond of collecting freaks in the way of specimens (especially if they have two or three extra dormer windows in their antra) to offset general rules than my friend Cryer, but this ransacking the whole country to run down freaks in order to prove that it is right to sacrifice these priceless jewels, the teeth, I do not believe in. I am trying in my humble way to prove to you that the sacrifice of teeth for the prevention or correction of malocclusion is wrong, is unnecessary, and that it is followed by such far-reaching and baneful effects that its practice should be abandoned, especially by the intelligent, conservative men of our profession. The "odontocides" should diminish in numbers, and they will diminish in numbers just in proportion as occlusion in all of its bearings is understood, and I dislike to hear even feeble efforts on the part of good men to defend a practice so pernicious. It is of the "old school" and not of the new, the scientific, the progressive in orthodontia. Now, I have shown you the worst cases that have ever come to my practice through the long busy years; in other words, I have put my theory to the severest tests, and in every single instance in all these cases we have found that both the occlusion and art requirements were best fulfilled by simply carrying out Nature's plan of putting all the teeth in their normal positions—normal occlusion.

There is no man in the dental profession for whom I have more respect and for whose work I have a higher admiration than for that of my friend Cryer, and I am surprised when he says we cannot push the molar teeth distally. I showed you several cases this evening where it had been done, and if you will come to St. Louis I will show you hundreds of cases where it has been done. This distal movement of molars has been in my daily practice ever since we have had the Baker anchorage, as it is, also, with my students and most of the members of the American Society of Orthodontists, and I have yet to see or learn of any symptom or result any more unfavorable than follows any other tooth movement.

Dr. Roberts spoke of a case where the lower jaw migrated mesially after the fifteenth year. I could not venture an opinion without first seeing the case, and probably even then I could not tell much about it, for there are many things about the third class that are still shrouded in mystery. I have been surprised with the ease with which some cases belonging to this class have been corrected, and equally surprised at the difficulties in treatment or even failures met with in other similar cases.

I am impressed by what Dr. Schamberg says in regard to the possible and probable influence that enlarged tonsils and adenoids have in causing some patients to protrude their lower jaws the better to provide for themselves breathing space in this class of cases.

I agree with Dr. William Trueman that we ought to know more about the cases we treat in after years. I am sure there would be revealed a large percentage of failures, but I also believe these failures could be traced to simple causes, either from following an incorrect line of treatment, or from improper methods of retention, and yet there are many, many cases treated that are lasting successes. I could show you hundreds. I did show you one on the screen to-night, but you know it is natural to be more interested in and enthusiastic over the cases we have just completed or that we are still working on. If Dr. Trueman, or any of you, will come to St. Louis, I will be pleased to show you many of them which I believe to be more of successes than your best fillings because they are more permanent. I am surprised to hear my friend Trueman at this day and age speak in such disparagement of the practice of orthodontia. Such advice as he says he gives his patients in regard to treatment might have been excusable twenty years ago.

Gentlemen, I am extremely grateful to you for the attention and interest you have manifested, as well as for your kind words of commendation, and may the interest awakened here this night be fruitful of great good in the uplifting of this beautiful and useful branch of science.

